# Electrical properties of graphene-metal contacts

**DOI:** 10.1038/s41598-017-05069-7

**Published:** 2017-07-11

**Authors:** Teresa Cusati, Gianluca Fiori, Amit Gahoi, Vikram Passi, Max C. Lemme, Alessandro Fortunelli, Giuseppe Iannaccone

**Affiliations:** 1 Dipartimento di Ingegneria dell’Informazione, Università di Pisa Via G. Caruso 16, 56122 Pisa, Italy; 2 University of Siegen, Hölderlinstrasse 3, 57076 Siegen, Germany; 3 RWTH Aachen University, Chair for Electronic Devices, Aachen, Germany; 4 CNR-ICCOM, Istituto di Chimica dei Composti Organometallici, Via G. Moruzzi 1, 56124 Pisa, Italy

## Abstract

The performance of devices and systems based on two-dimensional material systems depends critically on the quality of the contacts between 2D material and metal. A low contact resistance is an imperative requirement to consider graphene as a candidate material for electronic and optoelectronic devices. Unfortunately, measurements of contact resistance in the literature do not provide a consistent picture, due to limitations of current graphene technology, and to incomplete understanding of influencing factors. Here we show that the contact resistance is intrinsically dependent on graphene sheet resistance and on the chemistry of the graphene-metal interface. We present a physical model of the contacts based on ab-initio simulations and extensive experiments carried out on a large variety of samples with different graphene-metal contacts. Our model explains the spread in experimental results as due to uncontrolled graphene doping and suggests ways to engineer contact resistance. We also predict an achievable contact resistance of 30 Ω·*μ*m for nickel electrodes, extremely promising for applications.

## Introduction

Low and reproducible metal-graphene contact resistance *R*_*C*_ (i.e., smaller than 100 Ω × *μ*m) is an imperative requirement for the industrial adoption of graphene in electronics^1–3^ and for the adoption of other two-dimensional materials, which often rely on the use of graphene-metal interfaces^4^.

However, graphene contact fabrication technology is not yet mature and fully reproducible, and therefore a broad range of experimental values of *R*_*C*_ is found in the literature for the same metal^5–12^. Measurements of graphene-metal contact resistance for different metals (Cr, Ti, Cu, Au, Ni, Pd and Pt) via transfer-length and four-probe methods are strongly dependent on factors such as deposition temperature and process conditions, in addition to intrinsic factors such as metal work function, number of graphene layers, back-gate voltage^5^. In addition, photoresist residues are generally an issue that leads to high contact resistance in experimental devices. A reduced contact resistance has been reported in the case of contacts to graphene edges or defects, and has been attributed to stronger covalent bonding of graphene and metal or to a reduction of the bonding distance, which would entail a larger orbital overlap compared to van der Waals contacts^8, 10, 13–16^.

Theoretical work has provided insightful contributions into the physics of graphene-metal contacts^15–24^. In the model proposed by Xia *et al*.^17^, electrons first tunnel through the graphene-metal interface and then transfer from the graphene region under the metal to the graphene channel. Ji *et al*.^18^ used this concept in a systematic study of the contact resistance, including both single-sided and double-sided contacts for different graphene-metal systems. However, they compute graphene-metal tunnelling with the Wentzel–Kramers–Brillouin approximation, that is inadequate for high transmission, and their study is limited to a single geometry. Similar ab-initio studies only investigate lateral in-plane transmission, and therefore completely neglect the critical issue of vertical transport through the heterointerface^19–22^.

The contact geometry affecting the formation of covalent bonds at the interface certainly plays a role. Stokbro *et al*.^23^ showed that the nickel-graphene contact resistance is independent of the orientation of graphene and of the contact area, in agreement with experimental observations, but the actual resistance calculation is roughly approximated. Liu *et al*.^5^ further analysed the impact of molecular orbitals involved in the contact on transmission, finding that the conductance of the metal-graphene-metal junction is affected not only by the interfacial binding, but also by which molecular orbitals are involved and their symmetry, and that contact resistance decreases with the increase of the contact area at low bias voltage.

Ma *et al*.^24^ carried out a systematic first-principles study on contact resistance between several metals and graphene, observing the dependence of contact resistance on edge termination, contact area and point defects on the contact region. As in the pioneering work by Matsuda *et al*.^15, 16^, they identify three categories of graphene-metal contacts on the basis of the interaction strength. Metals weakly interacting with graphene (i.e. Au and Ag) are very sensitive to the atomistic configuration at the contact region: edges without chemical terminations, small contact length and point defects result in decreased contact resistance. Strongly interacting metals (i.e. Ni and Pd) show small sensitivity of the contact resistance to those factors. Finally, metals like Pt and Cu, with an intermediate strength of interaction, exhibit a slight dependence of the contact resistance on the details of the atomistic configuration of the contact. Matsuda *et al*.^16^ have shown that the different interaction strength can affect the contact geometry and give rise to “edge” configurations.

Finally, Barraza-Lopez *et al*.^20^ conducted an insightful investigation on graphene suspended between Al contacts and singled out charge transfer at the leads and into the freestanding section as a determining factor, but their study was limited to a single metal and did not pursue a stringent comparison with experiment. Such a comparison is in general lacking in previous theoretical work, especially rigorously translating the obtained results in terms of a model at a higher level of abstraction which can only allow comparison with experiments and full validation of the models.

For this reason, we have performed a comprehensive theoretical and experimental study of graphene-metal contacts. An in-depth theoretical investigation based on ab-initio simulations helps us in understanding the nature of the graphene-metal interface at the most fundamental level. This in turn enables us to develop a simple analytical model of the graphene-metal contact based on few parameters, and to devise ways of experimental validation. Finally, a large variety of different graphene-metal contacts were fabricated and experimentally tested in order to validate the main hypotheses of our model and approach. Edge contacts are not considered in the model, because the experimental structures are sufficiently large to assume the graphene edge under the metal to be infinitely far away from the actual graphene-metal edge.

## Theory

**Figure 1:**
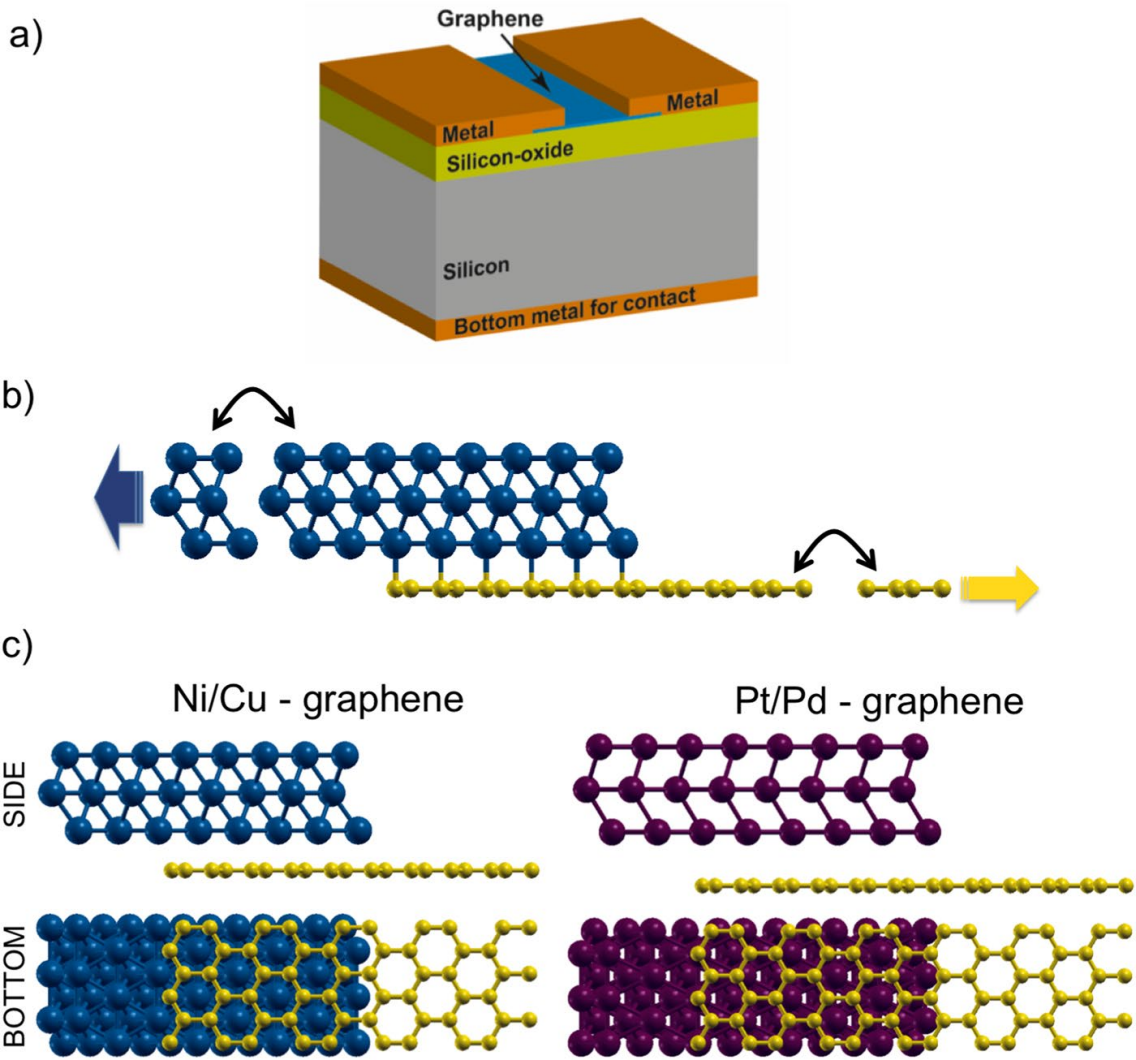
Graphene-metal interface. (**a**) Sketch of the graphene-metal contact with back-gate. (**b**) Schematic representation of the graphene-metal transmission model. Semi-infinite leads are attached on the left (metal) and on the right (graphene) of the simulated structure. (**c**) Side and bottom views of the scattering regions considered for the density-functional theory and transmission calculations, for the Ni-, Cu-, Pt- and Pd-graphene contacts.

We aim to gain insights into the main mechanisms at play when contacting graphene with different metals. To this end we investigate a specific, but realistic geometry of a graphene-metal interface, and perform a detailed analysis of the electrostatic potential at the graphene-metal interface. In Fig. 1, we show a sketch of the fabricated and simulated graphene-metal contact.

In order to properly consider transport across the graphene-metal heterointerface, as in ref. 5, 20, 23 and 24, we consider structures where electrons are injected from the metal on the left side or from graphene layer on the right side (Fig. 1(b)). We consider four different metals, divided in two categories, based on the binding energy and the graphene-metal distance: chemisorbed metals (Ni, Pd), with stronger bonds, and physisorbed metals (Cu, Pt), with weaker bonds (Fig. 1(c)).

**Figure 2:**
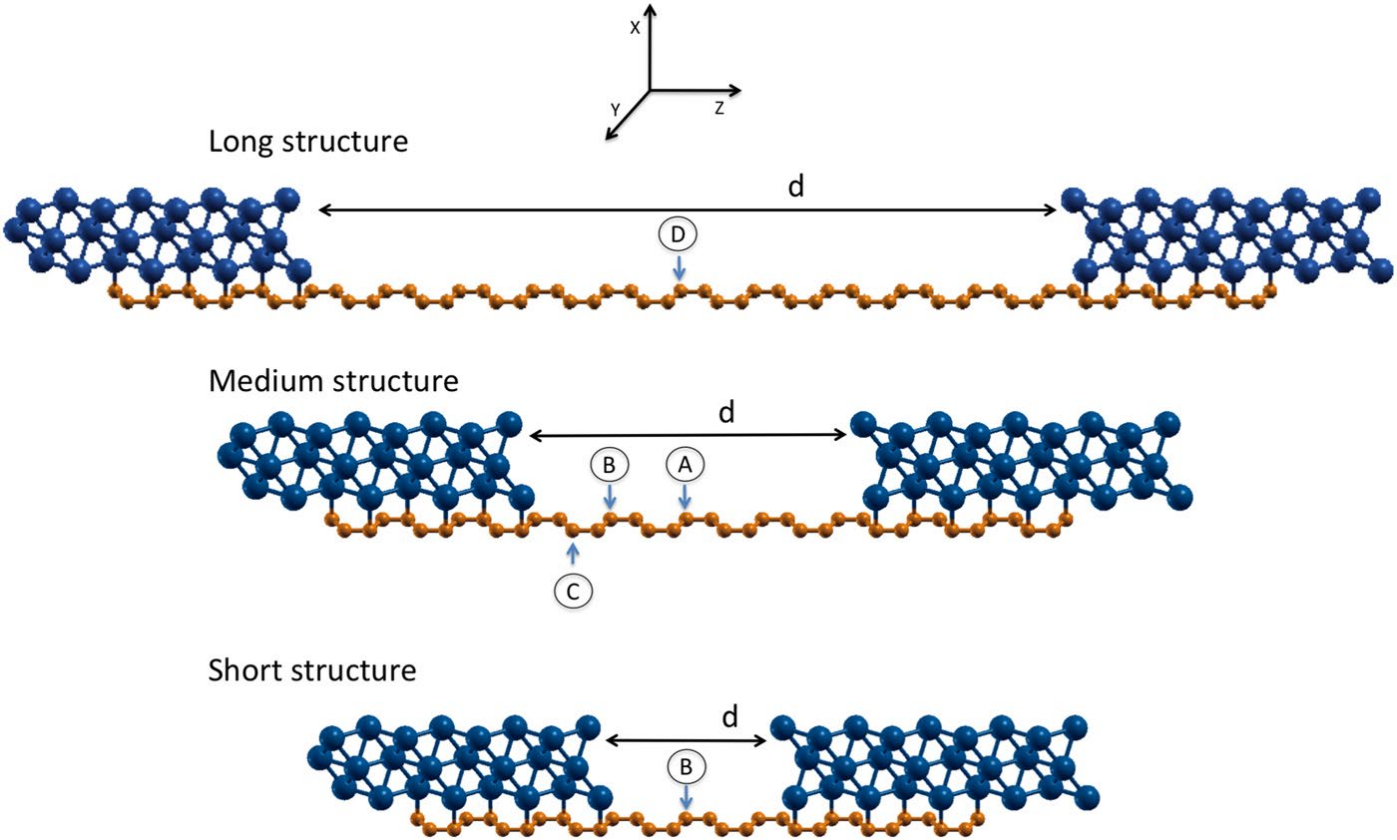
Structures investigated with ab-initio simulations include two metallic (nickel in the picture) regions connected by one graphene region of different lengths: a long structure (LS), where *d* = 4.53 nm for Ni/Cu systems and 5.02 nm for Pt/Pd systems (corresponding to a graphene region of 41 carbon atoms or 47 carbon atoms, respectively), a medium structure (MS), where *d* = 1.94 nm for Ni/Cu systems and 2.43 nm for Pt/Pd systems (corresponding to a graphene region of 17 carbon atoms or 21 carbon atoms, respectively), and a short structure (SS), where *d* = 1.08 nm for Ni/Cu systems and 1.57 nm for Pt/Pd systems (corresponding to a graphene region of 9 carbon atoms or 13 carbon atoms, respectively). Transport is computed by cutting the structure at one of the points indicated, and by attaching semi-infinite metal and graphene leads, as in Fig. 1(b), on the left and the right, respectively.

Our simulated structure (Fig. 2) is approximately symmetric in the *z* transport direction (approximately because of the ABC stacking) thus avoiding artefacts due to the presence of induced dipoles at the graphene-metal interface. Two metal islands are connected by a graphene sheet, placed in a side-contact configuration with the fragment geometries taken from separated metal and graphene components. The only free geometric parameter is the graphene-metal distance, which is optimised at the DFT level. In order to study the effect of the distance *d* between the two metal islands, three systems with different *d* have been considered, as illustrated and described in Fig. 2: a long structure (LS), a medium structure (MS), and a short structure (SS).

We perform first-principles DFT calculations with the Quantum Espresso package^25^, a plane wave basis set, a gradient-corrected exchange correlation functional (Perdew-Burke-Ernzerhof (PBE))^26^, and ultrasoft pseudopotentials (US-PPs)^27^ in scalar relativistic form, we include dispersion corrections (for more details see the Supporting Material) and we follow an analysis similar to that proposed in ref. 20.

To compute transport, we cut the system as illustrated in Fig. 1(b), considering for graphene three different cutting points as shown in Fig. 2 (indicated with A,B,C), and attach a semi-infinite metal lead to the left, and a semi-infinite graphene lead to the right. We then compute the transmission coefficient using the PWCOND module of Quantum Espresso^28, 29^.

The conductance *G* (and therefore the contact resistance *R*_*C*_ = *G* ^−1^) is obtained within the Landauer-Buttiker linear response theory^30, 31^

1 $$G={R}_{C}^{-1}=\frac{2{q}^{2}}{h}\int -T(E)\frac{\partial f}{\partial E}(\frac{E-{E}_{F}}{kT})dE$$

where *q* is the elementary charge, *h* is Planck’s constant, *k* is Boltzmann’s constant, *T* is the temperature, *T*(*E*) is the trasmission coefficient as a function of energy, *E*_*F*_ is the Fermi energy and *f* the Fermi-Dirac function.

Transmission is affected by two main factors^20^: (i) the evolution of the Dirac point energy *E*_*D*_(*z*) along the transport direction, which determines the number of electronic states available for transport (it should be recalled that in graphene the density of electronic states is zero at the Dirac point); (ii) the potential barrier and intermixing of orbital states at the interface between the two materials.

**Figure 3:**
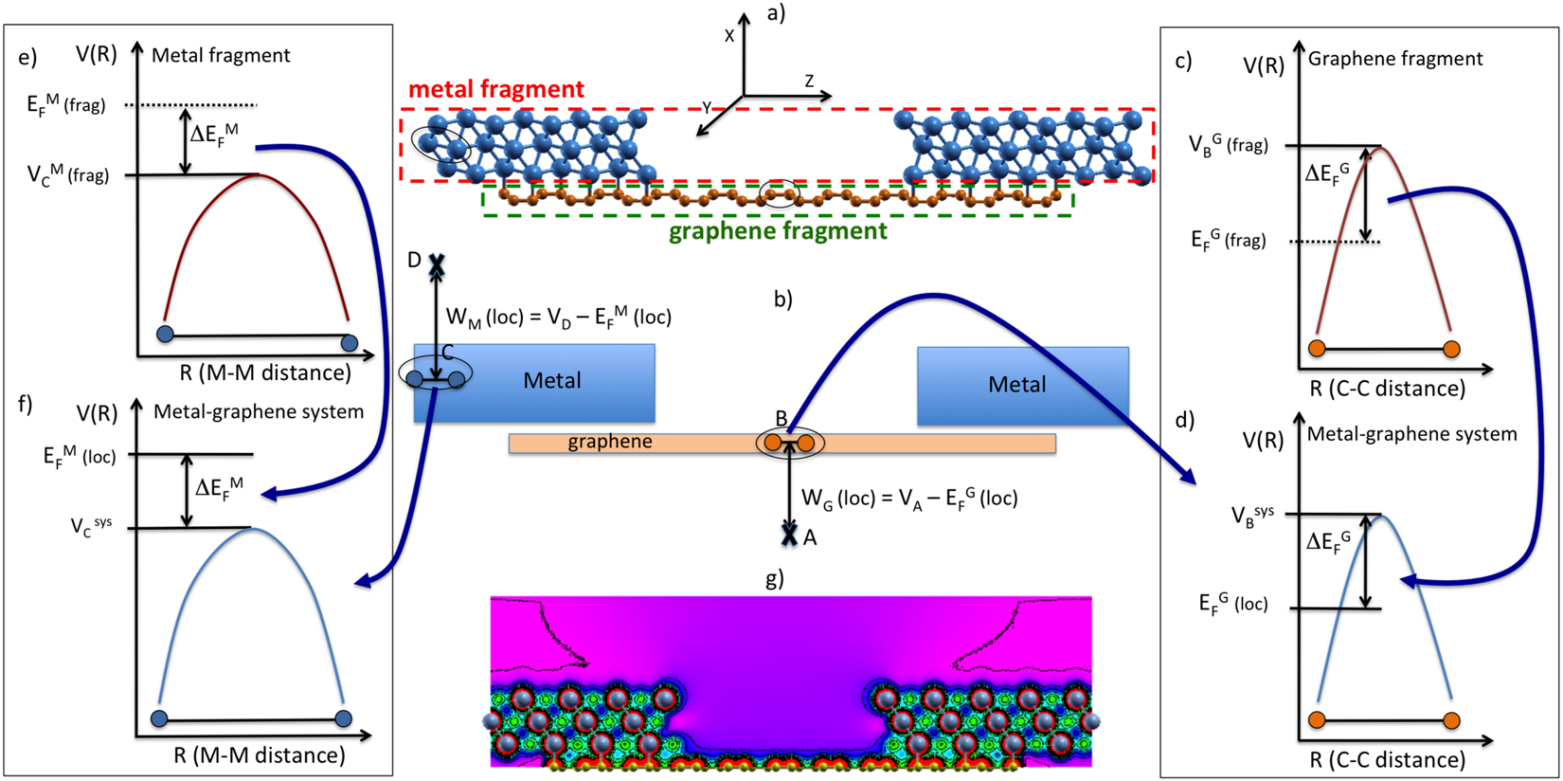
Analysis of the electrostatic potential in the case of the Ni medium structure. (**a**) Atomistic and (**b**) schematic representation of graphene-metal contact. The electrostatic potential (*V*(*R*)) analysis is also shown. (**c**) 1-D plot of *V*(*R*) between nearest neighbors carbon atoms of the graphene fragment; (**e**) 1-D plot of *V*(*R*) between nearest neighbors metal atoms of the metal fragment. From (**c,e**), the difference between the *V*(*R*) in the middle of the (**c**) *C*-*C* or (**e**) *M*-*M* bond and the Fermi energies of the fragments is extracted as ${\rm{\Delta }}{E}_{F}^{G}$ and ${\rm{\Delta }}{E}_{F}^{M}$, respectively. (**d,f**) 1-D plot of *V*(*R*) between nearest neighbors (**d**) carbon atoms or (**f**) metal atoms of the component system, respectively. Using the ${\rm{\Delta }}{E}_{F}^{G}$, ${\rm{\Delta }}{E}_{F}^{M}$ values from (**c,e**), the local Dirac point *E*_*D*_ (loc) and the local Fermi energy in the metal *E*_*F*_ (loc) are determined. Hence, the local Fermi energies are substracted to the appropriate vacuum levels to obtain in (**b**) the local Work Functions: *W*_*G*_ (loc) = *V*_*A*_ − *E*_*D*_ (loc) and *W*_*M*_ (loc) = *V*_*D*_ − *E*_*F*_ (loc) of graphene and metal components, respectively. (**g**) 2D-plot of *V*(*R*) on a (*x*, *z*) plane containing both metal and graphene atoms.

**Figure 4:**
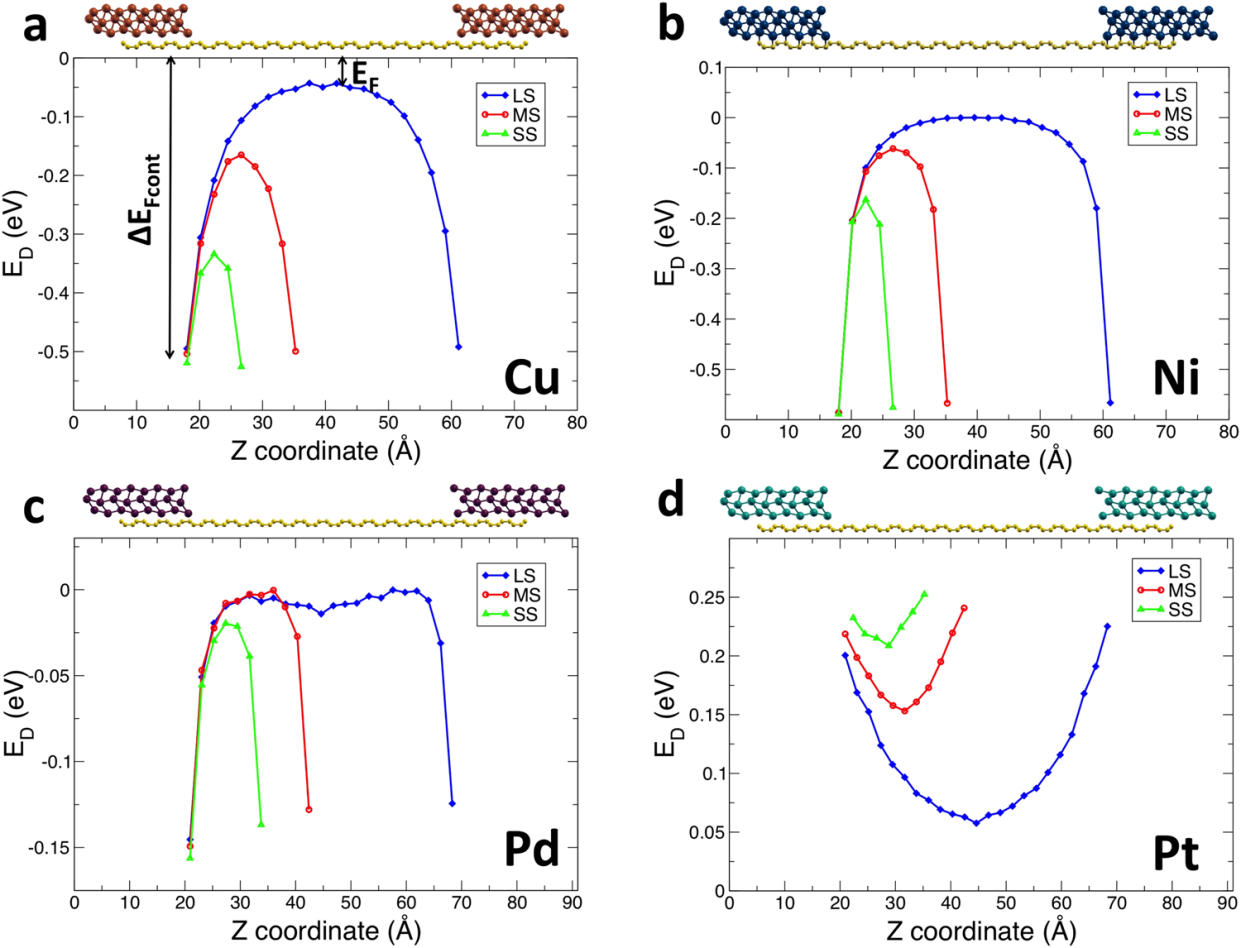
Dirac point energy profile. Profile of the Dirac point energy *E*_*D*_(*z*) as along the transport direction for all the considered metals and for different distances between the metal islands (long structure (LS), medium structure (MS), short structure (SS)). A picture of the long structure is shown at the top of each picture. In all cases the Fermi Energy is taken as zero.

We extract the energy of the Dirac point at each given point of the graphene in the interacting system from a comparison of the background electrostatic potential evaluated in the interacting system with the electrostatic potential in isolated graphene^32^. This procedure is illustrated in Fig. 3 using the medium structure as a working example; however, it is completely general and in Fig. 4 it is applied to the short and long structures as well. In detail, we first calculate the electrostatic potential between a pair of carbon or metal atoms as in points B and C of the interacting system in Fig. 3(b). We then calculate the electrostatic potential in the corresponding points in the isolated fragments: see Fig. 3(c) for graphene and Fig. 3(e) for the metal, respectively. By adding to the electrostatic potential at the given point in the interacting system the difference between the Dirac point (or the Fermi energy) and the electrostatic potential at the same point in the graphene (or metal) fragment, we obtain the position of the Dirac point (or Fermi energy) in the interacting system: see Fig. 3(c,d) for graphene and Fig. 3(e,f) for metal, respectively. Finally, by taking the difference of these local Fermi energies (i.e., Dirac point or metal Fermi energy) with respect to vacuum levels on the appropriate side of the system (point A for graphene and point D for the metal, respectively, in Fig. 3(b)) the local work functions for both graphene and metal, *W*_*G*_ (loc) and *W*_*M*_ (loc), respectively, are also derived as a by-product of this analysis. It is an important validation of the proposed procedure that the local work functions so estimated in the interacting system in points of the graphene or the metal far from the contact region coincide with those of the free fragments.

This analysis also allows us to obtain the complete profile of the Dirac point energy *E*_*D*_(*z*) along the longitudinal direction. In Fig. 4 we show the profile of *E*_*D*_(*z*) extracted from the electrostatic potential along the z-axis for *x* = *y* = 0 at the middle of carbon-carbon double bonds of graphene, for the short, medium and long structures (SS, MS, and LS, respectively), for the four considered metals. The curvature and the decay length of *E*_*D*_(*z*) are related to the sign and amount of charge redistribution at the interface. As apparent from the difference in curvature for the different systems, electrons are transferred from metal to graphene in the case of Cu, Ni, Pd but from graphene to the metal in the case of Pt. Moreover, also the decay length of *E*_*D*_(*z*) is very different for the different metals. This is due to an interfacial charge redistribution occurring also in the *z*-direction, determined by the interplay of charge donation from graphene to the metal and back-donation into the graphene electronic edge states^35, 36^.

The system thus develops a dipole moment along both *x* and *z* directions, which can be taken as useful descriptors of interfacial charge redistribution. The *x*-dipole is related to the graphene-metal charge transfer and the difference in electrostatic potential between graphene underneath the metal contact and bulk graphene, although the net result of this complex charge and electrostatic potential redistribution also depends on the change in the metal work function due to graphene adsorption^33^. It is important to note that such *x*-dipoles in our systems are rather different from those obtained on extended graphene-metal contact of identical geometry^33^, as reported in Table 1. The nanoscale character of the contact thus reflects on the features of charge injection (and therefore transmission) at the contact. Working with 3D periodic boundary conditions, such *x*-dipole is compensated by introducing a dipole correction^34^. The *z*-dipole instead is related to the other quantity affecting transmission as apparent from Fig. 4, i.e., the decay length of *E*_*D*_(*z*) from the contact.

**Table 1:** Perpendicular dipole moment for the interrupted and extended configurations of the systems under consideration (1 Debye = 3.336 × 10^−30^ C · m).

Material	Interrupted structure	Infinite structure
	Perpendicular Dipole (D)	Perpendicular Dipole (D)
Cu	−0.34	−0.32
Ni	−1.09	−0.49
Pd	−0.90	−2.60
Pt	−1.49	−2.68

We merge the effects of the dipole in the *x* and *z* directions into a single quantity: Δ*E*_Fcont_, i.e. the difference between the Fermi energy and the Dirac point energy at the graphene-metal interface (illustrated in Fig. 4(a)). Δ*E*_Fcont_ depends on the metal species and on the contact geometry: in the case of Ni it is almost insensitive to structural features, whereas in the case of Pd it changes sign from positive (p-doped) to negative (n-doped) at a distance between the Pd plane and the graphene plane of about 2.56 Å. We then use this quantity in the analysis of computational results in the next section.

## Results and Discussion

We consider two different systems (MS and SS) and different cut points to which we apply the semi-infinite graphene lead (indicated with A, B, C in Fig. 2).

**Figure 5:**
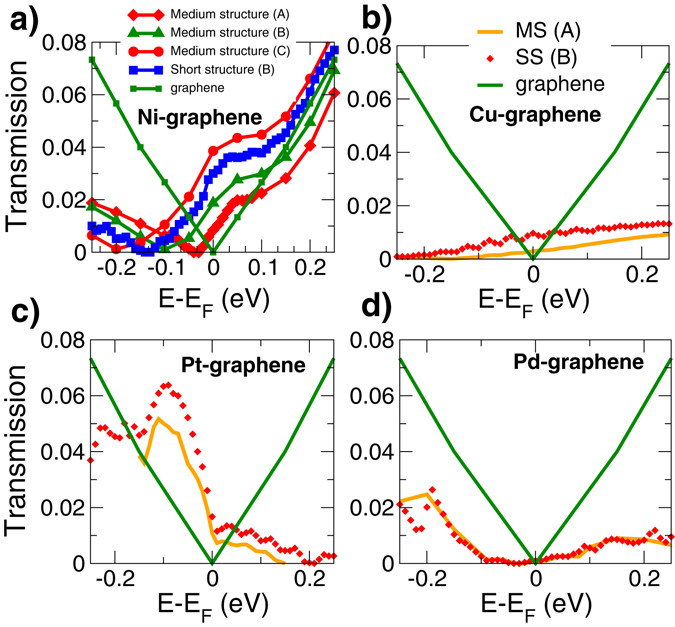
Transmission simulation of different contacts. (**a**) Transmission coefficient as a function of energy obtained with PWCOND for nickel-graphene contacts for different cutting points, corresponding to those indicated in Fig. 2 and compared with the transmission of the ideal graphene monolayer (green diamonds). It is clear that the transmission coefficient and the conductance strongly depend on the cut point. (**b–d**) Transmission for graphene-metal systems obtained with PWCOND for different metals (Cu,Pd,Pt) compared with the transmission of the ideal graphene monolayer (in green), for the short structure at point B (SS  B, in red) and for the medium structure at point A (MS A, in orange).

As can be seen in Fig. 5, where *T*(*E*) is shown as a function of the energy for different metals, the main impact of the structure (MS or SS) and of the choice of the cut point on the profile of *T*(*E*) is a shift in energy: T(E) is zero for the Dirac point energy of the cut point, which corresponds to zero density of electronic states in the graphene semi-infinite lead. The graphene Dirac-point energy moves to the Fermi energy as one moves away from the contact, because graphene is undoped and the electric field is progressively screened (Fig. 4).

On the other hand, the resistance *R*_*C*_ of the medium structure and of the short structure are very different, because they depend on the transmission coefficient in the vicinity of the Fermi energy, as summarised in Table 2. Such large dependence of the resistance on the potential profile in close proximity of the contact can explain the wide variation in experiments and in theoretical results in the literature. Indeed, the contact resistance clearly depends on the Dirac point energy in graphene, and therefore on the charge density in the graphene layer, which is often neither reported nor controlled in the experimental literature. Let us stress here that the length scale at which the Dirac point energy changes is very small (less than 2–3 nm from the metal surface), therefore four-probe or transfer-length methods cannot correct for this effect in the contact resistance. It is an uncontrolled factor, up to now, but very important: even in the case of ballistic transport, graphene resistance per unit width strongly depends on Dirac point energy, reaching a minimum of about 310 Ω · *μ*m.

**Table 2:** Contact resistance per unit width (*R*_c_). For different metals, we compare results of our simulations for the medium (point A) and short (point B) structures, with experimental and theoretical results from the literature.

Material	Medium structure	Short structure	Experimental Results	Theoretical results
	(Ω · *μ*m)	(Ω · *μ*m)	reported (Ω · *μ*m)	reported (Ω · *μ*m)
Cu	1153	374	184–263^43^	627^18^
			92–254^46^	44^19^
Ni	360	120	294^12^, 300^37^	600^23^
			800^38^	150–350^40^
Pd	2899	2076	320–715^9^	403^18^
			600^37^	
			185–230^17^	
			584^43^, 122–484^46^	
Pt	209	123	—	764^18^

We should also notice that our results for palladium-graphene contacts are far from experiments. Our interpretation is that for Pd-graphene we consider a contact interface that is rather different from the experimental one, and further investigation is required to properly capture the interface chemistry. In detail, palladium is known to easily mix with carbon to produce carbide phases^10^, thus completely altering the atomistic structure of the interface, a phenomenon which is not expected to occur in the case of Ni, Cu or Pt electrodes.

On the basis of these observations, we can devise a simple physical model of the contact, which depends on an effective transmission coefficient that is a function of the metal and of the interface geometry, and on the Dirac point energy in the graphene layer between the two metal islands.

Indeed, in Table 3, we propose a simple analysis of transmission results: for all metals and for the short and medium structures, we consider the contact resistance *R*_*C*_ and the ballistic resistance of the semi-infinite graphene lead (“Graphene R” column), which is computed as in Eq. (1) considering a single analytical expression for T(E)^39^ and which only depends on the difference between the Fermi energy and the Dirac-point energy at the cut point (Δ*E*_*F*_), that is the symmetry point of the structure (point *A* for medium and point B for short structures in Fig. 2, respectively).

**Table 3:** Analysis of the simulation results for the contact resistance. Ballistic resistance of graphene in the semi-infinite lead applied to the symmetry point (point A or B for medium and short structures, respectively) of the structure (Graphene R) and resistance of the graphene-metal contacts (*R*_c_) for different metals and length of the simulated structure. T_eff_ is the ratio of Graphene R to Total R and depends on the metal species but not on the length of the structure.

Metal	Short structure				Medium structure			
	*R*_*C*_ (Ω · *μ*m)	Δ*E*_*F*_ (eV)	Graphene R (Ω · *μ*m)	*T*_eff_	*R*_*C*_ (Ω · *μ*m)	Δ*E*_*F*_ (eV)	Graphene R (Ω · *μ*m)	*T*_eff_
Cu	374	−0.336	46.6	0.12	1153	−0.166	70.6	0.06
Ni	120	−0.136	83	0.69	360	−0.036	245	0.74
Pd	2076	−0.036	230	0.11	2899	−0.025	273	0.12
Pt	123	0.209	58.3	0.47	209	0.155	77	0.44

We can see that the ratio *T*_eff_ = *R*/*R*_*C*_ is practically the same for the MS and the SS, and only depends on the type of metal and of the contact interface. The ratio *T*_eff_ can be interpreted as an effective transmission coefficient at the Fermi energy, which is an intrinsic property of the graphene-metal interface, and that can also be extracted from *T*(*E*) shown in Fig. 5

2 $${T}_{{\rm{eff}}}=\frac{R}{{R}_{C}}=\frac{T({E}_{F})}{[\frac{\mathrm{2|}{E}_{F}-{E}_{D}|}{\pi \hslash {v}_{F}}]},$$

where *v*_*F*_ is the Fermi velocity in graphene and the denominator between square brackets is the ballistic transmission coefficient of a graphene sheet^39^.

Since interband tunnelling in graphene is favoured by the absence of a gap and does not limit transmission, the other relevant parameter for the measured contact resistance is the Dirac-point energy in graphene at the cut point $\Delta {E}_{F}=|{E}_{F}-{E}_{D}|$, that in practical cases means at the distance of few decay lengths (very few nm) from the interface.

We therefore have defined a simple physical model of the contact — even simpler than that described by Chaves *et al*.^40^ — based on only two parameters that can be extracted through ab-initio simulations: *T*_eff_ and Δ*E*_Fcont_. The value of Δ*E*_*F*_ depends on the electrostatics of the structure or of the actual device which the contact is part of.

In detail, we can express *R*_*C*_ as:

3 $${R}_{C}={[{T}_{{\rm{eff}}}\frac{2{q}^{2}}{h}\int -\frac{\partial f(\frac{E-{\rm{\Delta }}{E}_{F}^{\ast }}{kT})}{\partial E}\frac{\mathrm{2|}E|}{\pi \hslash {v}_{F}}dE]}^{-1}$$

where $${\rm{\Delta }}{E}_{F}^{\ast }=\,{\rm{\min }}({\rm{\Delta }}{E}_{{\rm{Fcont}}},{\rm{\Delta }}{E}_{F})$$.

Our model predicts that the measured contact resistance is a linear function of the graphene sheet resistance in the case of diffusive transport, when both are extracted using the transfer-length method on a relatively large structure (both quantities have the same dependence on Δ*E*_*F*_). This prediction can be used as a means to validate or to falsify the model. Let us highlight that experiments in the literature^17, 46^, have shown the dependence of graphene-metal contact resistance on the back-gate voltage (and therefore on Dirac-point energy), but have never pointed to the mentioned linear dependence and to the related physical model.

## Experiments

**Figure 6:**
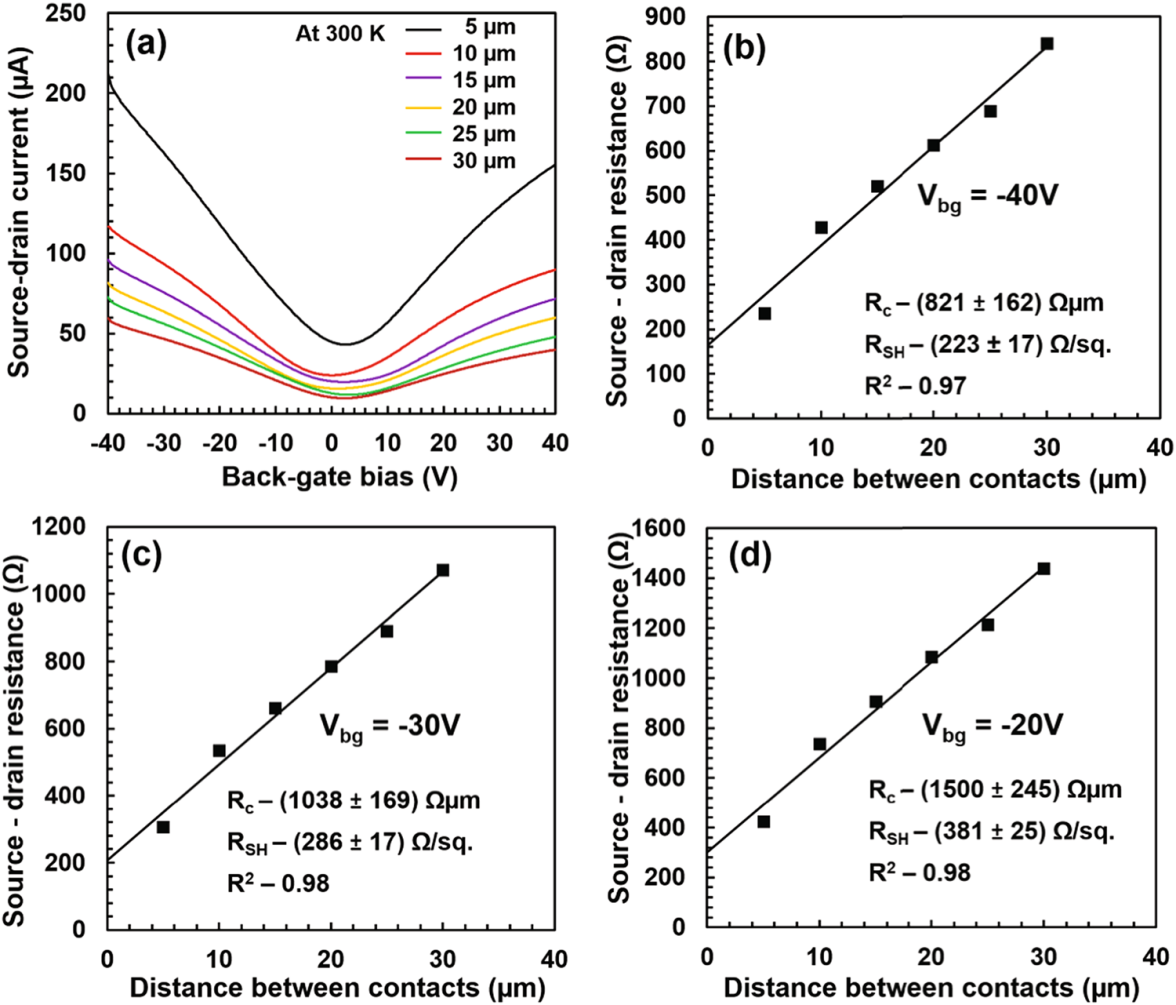
Experiments on graphene-nickel/gold contacts. (**a**) Transfer characteristics of a TLM structure with nickel/gold contacts on graphene as a function of the back-gate voltage *V*_*bg*_ for different spacing between contacts. Temperature is 300 K and the voltage applied between the contacts is 50 mV. (**b–d)** Total resistance between source and drain contacts as a function of spacing between contacts for back-gate voltage *V*_*bg*_ of −40 V (**b**), −30 V (**c**), −20 V (**d**). Squares are experimental data. From the least mean square fit (line), the contact resistance *R*_*C*_ from the Y-intercept and the sheet resistance *R*_sh_ from the slope of the line are extracted.

P-doped silicon wafers with a boron concentration of 3 × 10^15^ cm^−3^ and with thermally grown silicon dioxide of 85 nm were used as starting substrates. Samples of 13 mm × 13 mm were diced from the entire wafer. Graphene was transferred to the chips and etched to from device structures. Different metals were deposited to form electrical contacts. Details of this process can be found in the Methods section. Metals used for the study include copper (150 nm), gold (150 nm), nickel/gold (25 nm/125 nm), palladium (150 nm), platinum/gold (25 nm/125 nm). Spacing between the contacts is varied from 5 *μ*m to 30*μ*m in order to extract both the contact resistance and the sheet resistance using the Transfer-Length Method (TLM). Electrical measurements were carried out on TLM structures under a varying back-gate bias *V*_*bg*_ which enables tuning of the Dirac point energy (shown in Fig. 6(a–d) for the nickel-graphene contact and in the Supplementary Information for the other metal contacts).

**Figure 7:**
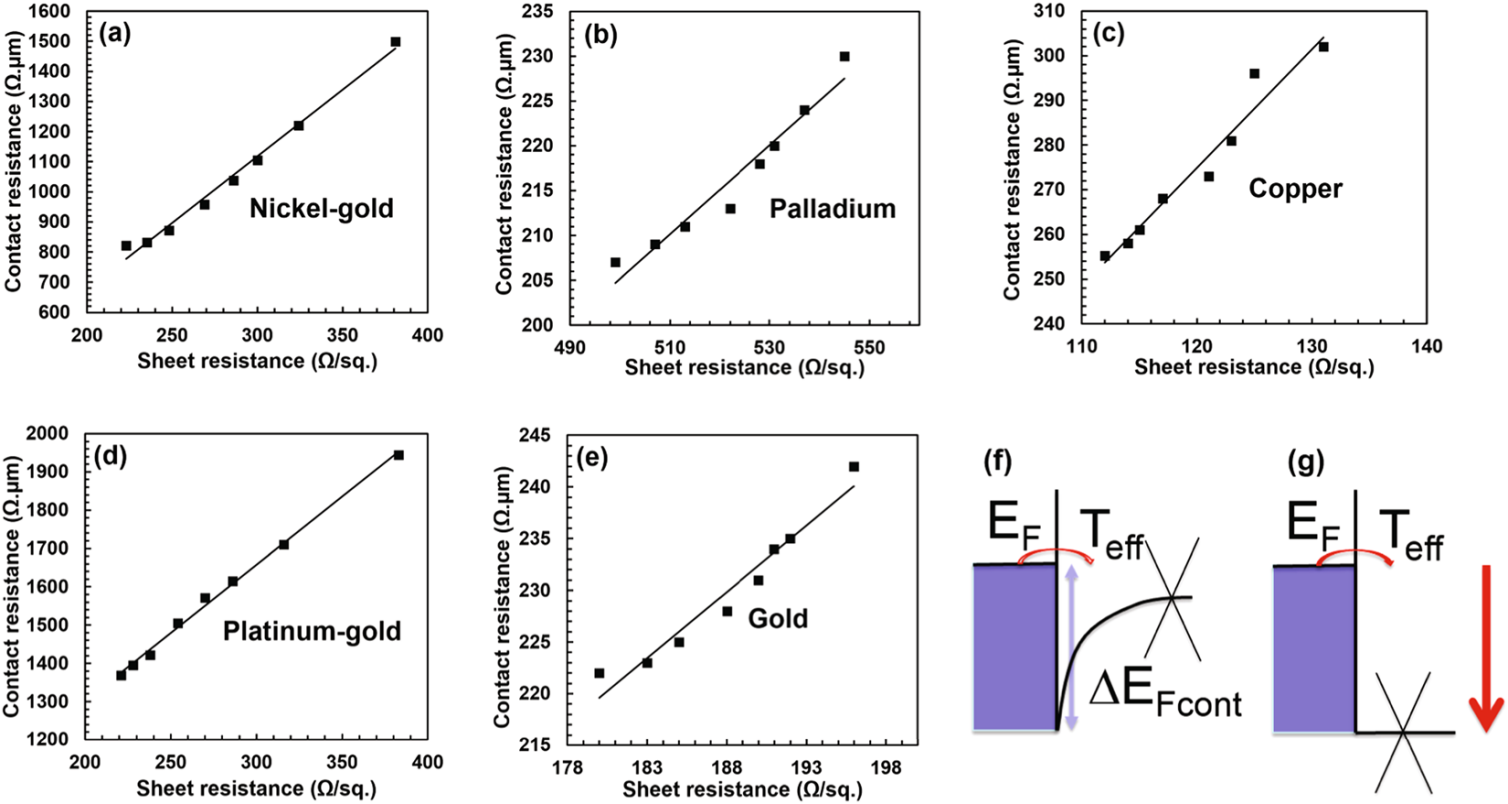
(**a–e**) Experiments: Plots of contact resistance vs. graphene sheet resistance for five different graphene-metal contacts (nickel/gold (**a**), palladium (**b**), copper (**c**), platinum-gold (**d**), gold (**e**)). Each point corresponds to a different back-gate voltage *V*_*bg*_ (−40 V, −39 V, −37 V, −33 V, −30 V, −28 V, −25 V, −20 V). In all cases the correlation coefficient of the linear fit is very high (*R* > 0.96), confirming our interpretation. (**f**) Illustration of the simple model of graphene-metal contact and (**g**) condition of minimum contact resistance, obtained by properly adjusting the graphene Dirac point near the contacts.

In Fig. 7(a–e) we show, for each type of metal, a scatter plot of the contact resistance extracted with the transfer-length method versus the measured graphene sheet resistance, where each point is obtained for a different back-gate bias from −40 V to −20 V. As can be seen, the correlation coefficent of the linear fit between the two quantities is very high (R > 0.96), validating our interpretation and our proposed model.

The absolute values of contact resistance are in reasonable agreement with our simulations, but they depend on the details of the contact interface which are not fully known from experiments. However, the most important result is the functional relation between contact resistance and sheet resistance, that is fully proven.

## Conclusion

We have obtained a in-depth understanding of graphene-metal contacts, based on detailed ab-initio simulations of the electrical properties of graphene-metal structures, and carried out a systematic experimental investigation of different metal contacts and different geometries. We have derived and validated a simple model which captures the relevant physics and is based on only two parameters: an effective contact transmission coefficient *T*_eff_ and the difference between the Fermi energy and the Dirac-point energy at the interface Δ*E*_Fcont_ (Fig. 7(f)).

Since the screening length in graphene is only 1–4 nm, both the transfer-length and four-point methods cannot eliminate the effect of graphene in the vicinity of the contact on the measured contact resistance. Therefore, as we have demonstrated experimentally, the measured contact resistivity must be linearly dependent upon the graphene sheet resistance. Absolute predicted values for the contacts resistivity follow similar trends as experimental data, except in the case of highly reactive metals. The latter are likely to form alloys with graphene, which is not yet incorporated in our model.

From this understanding we can draw as one main conclusion that we can optimise the contact resistance by adjusting the Dirac-point energy of graphene near the metal via the back-gate voltage (Fig. 1(a)), thus tuning the system into the condition illustrated in Fig. 7(g), i.e. with flat potential in the graphene layer in the contact region, so that we have minimum sheet resistance of graphene close to the contact. In a practical industrial application, the same result could be obtained with suitable doping. To translate this conclusion into quantitative values, in Table 4 we indicate the energy of the Dirac point at the contact with respect to the Fermi energy, and *T*_eff_ obtained from the medium structure. The minimum graphene resistance *R*_Gmin_ is the ballistic resistance per micron width in the case of flat potential for the Dirac-point energy indicated in the second column. Then, the minimum (asymptotic) contact resistance is obtained as *R*_Gmin_/*T*_eff_. As can be seen, contact resistance can be as low as 30 Ω · *μ*m for nickel contacts, which would be very desirable for high performance graphene-based FETs^44, 45^.

**Table 4:** Evaluation of minimum achievable contact resistance. The minimum asymptotic contact resistance achievable for different metals in the situation illustrated in Fig. 7(f) is shown in the last column. We have considered T_off_ and *E*_Fcont_ extracted from ab-initio simulations on the medium structure. *R*_Gmin_ is the ballistic resistance of graphene per unit width for the corresponding *E*_*F*_ − *E*_Fcont_. The contact asymptotic resistance is obtained as *R*_Gmin_/*T*_eff_.

Metal	*E*_*F*_ − *E*_Fcont_ (eV) interface	*T*_eff_ interface	*R*_Gmin_ (ballistic) (Ω · *μ*m)	Minimum achievable *R*_*C*_ (ballistic) (Ω · *μ*m)
Cu	0.583 (n-type)	0.12	23.5	196
Ni	0.559 (n-type)	0.69	21	30
Pd	0.171 (n-type)	0.11	68	618
Pt	−0.218 (p-type)	0.47	54	115

## Methods

The process steps of fabrication started with the cleaning of the samples in bath of acetone to remove photoresist used as a protection layer during dicing, followed by iso-propanol and de-ionized water rinse. Graphene growth on copper foil was performed in a NanoCVD rapid thermal processing tool using the method described in ref. 41 Poly-methyl-metacrylate (PMMA) which acts as a mechanical support layer was spin-coated on the copper foil. The grown graphene was transferred onto the silicon-oxide/silicon substrate using the electro-chemical delamination method^42^. After the transfer, the PMMA support layer was removed by immersing the sample in a bath of acetone overnight and successively annealing the sample for one hour in an atmosphere consisting of argon (95%) and hydrogen (5%) at a temperature of 450 °C. A positive tone photoresist was spin-coated on the substrate at a speed of 2500 rpm for 50 s and soft baked at 110 °C for 90 s to achieve a thickness of 1.4 *μ*m. Photolithography was done to define patterns on the resist followed by an oxygen (O_2_) plasma etch to etch graphene in the unprotected region and photoresist was removed by placing the sample in a bath of acetone overnight. At this step the channel region of the devices is defined. Using the same photoresist but in an image reversal lithography process, source-drain contacts were defined. Various metals were deposited on different samples by evaporation, followed by removal of excessive metal using lift-off process in AZ 100 solution which is kept at 70 °C.

Using a three probe configuration the total device resistance (of a single device) is measured by applying a varying back-gate voltage and a fixed voltage between drain and source (50 mV). This measurement procedure is repeated for varying contact separation and the total device resistance (Ω) is plotted as a function of the contact separation (*μ*m). A linear fit of the data is performed and the contact resistance *R*_*C*_ and sheet resistance *R*_sh_ are extracted. By multiplying the extracted values by the channel, width the contact resistance in Ω μm and sheet resistance in Ω/□ are obtained. The extracted values give a ± error value (taking the upper limit and the lower limit while fitting the scattered data points for extracting *R*_*C*_ and *R*_sh_) which is seldom reported in the literature (only in ref. 17).

## Electronic supplementary material

Supporting information
