# Supplementary material for: Electrical properties of graphene-metal contacts
Source: Sci Rep. 2017 Jul 11;7:5109. doi: 10.1038/s41598-017-05069-7 (PMC5506027; doi:10.1038/s41598-017-05069-7)
Supplement: Supplementary file 1 — Supporting information [file 41598_2017_5069_MOESM1_ESM.pdf]

Supporting information for: Electrical properties of graphene-metal contacts

Teresa Cusati,[†] Gianluca Fiori,[†] Amit Gahoi,[‡] Vikram Passi,[‡] Max Lemme,[‡] Alessandro Fortunelli,[¶] and Giuseppe Iannaccone[*,†]

[†]*Dipartimento di Ingegneria dell'Informazione, Università di Pisa*
Via G. Caruso 16, 56122 Pisa, Italy,

[‡]*University of Siegen, Hölderlinstrasse 3, 57076 Siegen, Germany, and*

[¶]*CNR-ICCOM, Istituto di Chimica dei Composti Organometallici*
Via G. Moruzzi 1, 56124, Pisa, Italy

E-mail: g.iannaccone@unipi.it

Phone: +39 050 2217677

DFT calculations

Electron transport has been studied in a system consisting of a scattering region attached to semi-infinite electrodes. We have performed first-principles DFT simulations using Quantum Espresso package[S1]. These calculations produce the one-electron energies and wavefunctions which are the basis for the transmission calculations using PWCOND[S2,S3] module of Quantum Espresso. It is assumed that the electrons move ballistically in the self-consistent potential with reflection and transmission restricted to the scattering region. In order to

[*]To whom correspondence should be addressed
[†]Università di Pisa
[‡]University of Siegen
[¶]CNR

calculate the contact resistance, the transmission coefficient has to be computed through the solution of the scattering problem between incoming and outgoing Bloch waves (for details see the references[S2,S3]). $T(E)$ is obtained as

$$T(E) = \sum_{\boldsymbol{k}_{\|}} T_{\boldsymbol{k}_{\|}} \left(\boldsymbol{k}_{\|}, E\right) = \sum_{\boldsymbol{k}_{\|}} \sum_{i,j} T_{i,j} \left(\boldsymbol{k}_{\|}, E\right), \tag{1}$$

where $T_{i,j}\left(k_{||}, E)\right)$ is the probability that an electron with energy E and transverse momentum $k_{||}$ incoming from the i-th Bloch state is transmitted to the outgoing j-th state of the other electrode. Sums run on both spins. The first sum is performed over $k_{||}$ belonging to the two-dimensional Brillouin zone (2D-BZ) of the supercell. This formalism is appropriate when all the system parameters, including magnetization, can be treated as static. We assume that electrons conserve quantum coherence and that reflection and transmission is only due to the self-consistent potential in the scattering region, with no additional scattering mechanisms.

Simulated systems

The simulated structure for the different metallic contacts are shown in Figure S1. Three regions are highlighted: the left (LL) and the right (RL) leads and the so-called scattering region (SR, corresponding to the central part). Left and right leads are ideally connected to semi-infinite bulk metal leads (left side) and graphene monolayer (right side).

The left lead is simulated as the semi-infinite repetition of a slab containing 3 atomic (111) crystalline planes (2 atoms per plane). We have chosen three layers per slab, which is a good trade-off between accuracy and computational effort. We have verified that results do not quantitatively change if a larger number of metallic layers is considered, as also indicated by previous theoretical works[S4–S6]. The right lead is simulated as the semi-infinite repetition of a slab containing 4 atoms of carbon per repetition unit.

The scattering region consists of an interrupted graphene monolayer and a metal step,

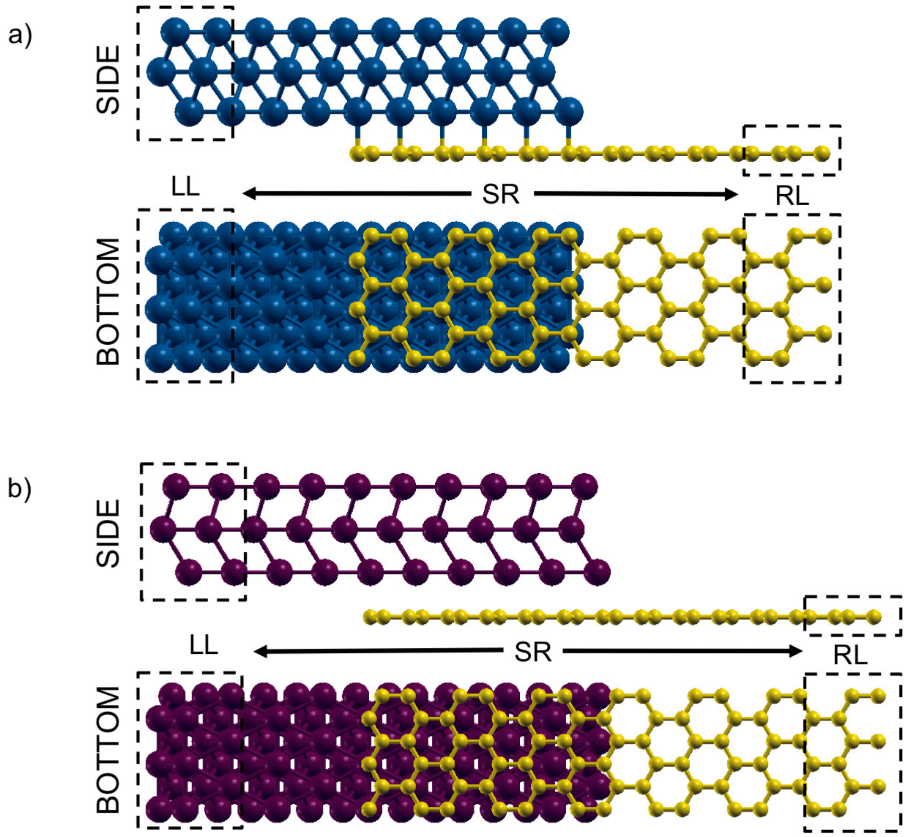

Figure S1: Atomic representation of the metal-graphene contact considered in the transport simulation: (a) Ni- and Cu-graphene contact and (b) Pt- and Pd-graphene contact. Left lead (LL) corresponding to metal, right lead (RL) corresponding to graphene and scattering region (SR), corresponding to the metal-graphene contact, are highlighted

composed by 8 atoms of metal/layer and 19 atoms of carbon for Ni and Cu systems and 23 for Pt and Pd systems, 10 and 14 of them, for Ni/Cu sytems and Pt/Pd systems respectively, are located in the metal-graphene contact region (overlap metal-graphene contact length = 10.79 Å and 12.65 Å for Ni/Cu and Pt/Pd respectively). For interrupted graphene monolayer, we mean a non-continuous graphene layer with interrupted periodicity on the left side of the cell. The orientation of the graphene edge is chosen to be zigzag, as it is energetically more stable[S7].

Periodic boundary conditions are assumed in y and z directions for electronics calculations. Since the cell corresponding to the scattering region is very large along x (vacuum) and z (transmission) directions (z=54.67 Å for Ni/Cu contact and z=65.53 Å for Pt/Pd one), the

BZ are sampled at k_x=k_z=1, while in the y direction convergence requires 12 k points (2.49 Å). The BZ of the right and left leads has been sampled using a 1x12x8 Monkhorst-Pack grid. Such BZ sampling has been checked for accuracy and found to be sufficient for obtaining a converged self-consistent potential needed for subsequent transmission calculations.

An orthorhombic cell was used, which allows us to obtain a uniform and stable configuration of the metal-graphene contact scattering region, adjusting the metal lattice size to that of graphene, and avoiding particular cuts on the borders of the metal steps that could arise if another cell (for instance an hexagonal one) was used.

Graphene lattice size is 2.49 Å. For Ni we have used the same lattice constant. Metal lattice have been strained to fit onto the graphene unit cell for the case of Cu, Pd and Pt. For Cu and Ni, the most stable configuration corresponds to one carbon atom on top of the metal atom and the second carbon on a hollow site. In the case of Pd and Pt, constant volume compression was applied, ensuring the lattice match in one of the coordinates (y direction) and adjusting the other two (x and z directions) by the same factor. When adjusting metal lattice size onto the fixed graphene lattice, the mismatches found were: Cu = 2.8 % and Pt = 10.5 % / Pd = 10.3 % in the x and z direction.

Vacuum regions of about 23 Å in the x direction for all systems are included to minimize the interaction between adjacent image cells. Also for this SCF calculations, a dipole correction is applied to avoid spurious interactions between periodic images of the slab[S8,S9]. Moreover, the configuration was constructed in the most symmetric way as possible, to avoid introducing artifacts in the electrostatic potential analysis, by the induction of dipoles.

An energy cutoff of 40 Ry is used for selection of the plane-wave basis set for describing the wave function and 400 Ry for describing the electron density cutoff. For the transmission calculations, we have used 301 k-points in the y direction and one k-point in the x direction.

The geometries of graphene and metal step were kept fixed and only the metal-graphene equilibrium distances were optimized. In order to determine the equilibrium distance to be used in our simulations for each system, we have performed an SCF scan series of calculations

of the scattering region, varying the metal-graphene distance in order to obtain the corresponding interaction potential, and thus to get the equilibrium distance. Dipole correction and the dispersion effects (Van der Waals corrections[S10]) were included in these simulations, which are important to determine the geometry of metal-graphene contacts. The results obtained are shown in Table S1.

Table S1: Equilibrium distance between the graphene and metal island fragments (d_{eq}, in Angstrom (1 Å= 0.1 nm)

System	d_{eq} (10^{-10} m)
Cu	0.27
Ni	2.10
Pd	2.80
Pt	3.00

Being Cu and Pd the systems most sensitive to geometry, and the ones with the lowest contribution to the trasmission, special attention was payed on the equilibrium distances used in both cases. We have carried out some studies related to the variation of d_{eq} between metal and graphene, taking into account some values reported in the literature previously[S8,S11]. In the case of Cu, there is no a significantly change in the transmission and consequently on the contact resistance (Fig. S2), with the change in d_{eq}, even if a small change in the ΔE_F is produced when the d_{eq} change. In contrast, Pd system shows a change in the transmision and ΔE_F values, changing its sign (going from p-doped to n-doped) with the diminishing of d_{eq}. In the energy range around $E - E_F$=0, no important variations are observed. The change in ΔE_F can be associated to the compression of the charges at the interface between the graphene and metal contact when the distance between both elements becomes small, for a metal that interacts strongly with graphene. Moreover, in the case of Pd, we conjecture that transmission is strongly affected by the formation of carbides at the interface with the carbon atoms of the graphene[S6,S12,S13].

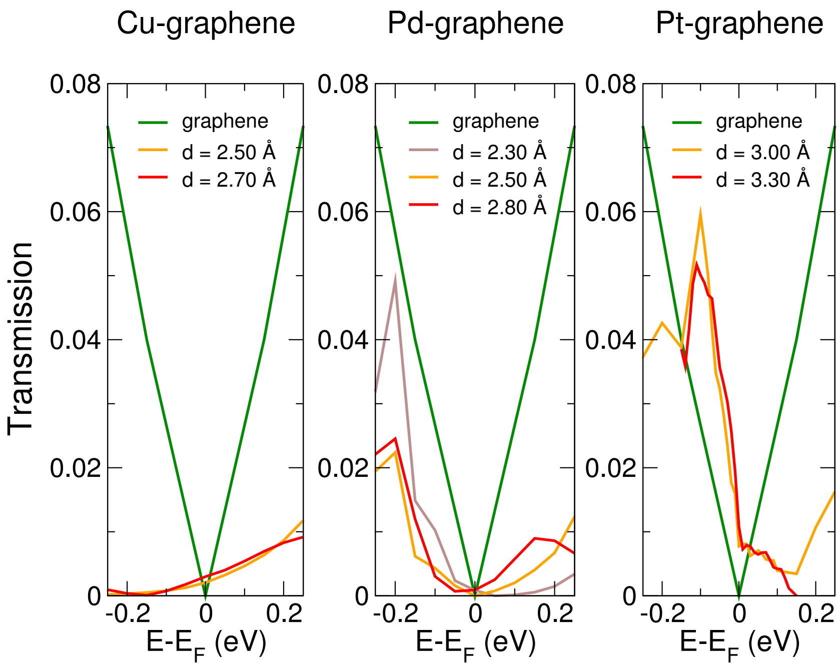

Figure S2: Transmission for Cu- (left panel), Pd- (middle panel) and Pt-graphene junction (right panel) for the long structure, considering two equilibrium distances in each case.

Transfer-length method experiments

We used the transfer-length method to extract the contact resistance and the sheet resistance for the various metals. Results for contacts between nickel-gold and monolayer graphene have been already reported in the main text (Figure 5a-d). Below we report results for palladium (Fig. S3), platinum/gold (Fig. S4), copper (Fig. S5), and gold (Fig. S6) in contact with monolayer graphene.

References

(S1) Giannozzi, P.; Baroni, S.; Bonini, N.; Calandra, M.; Car, R.; Cavazzoni, C.; Ceresoli, D.; Chiarotti, G.L.; Cococcioni, M.; Dabo, I. et al.; QUANTUM ESPRESSO: a modular and open source software project for quantum simulations of materials *J. Phys.: Condens. Matter* **2009**, 21, 395502

(S2) Smogunov, A.; Dal Corso, A.; Tosatti, E.; Ballistic conductance of magnetic Co and

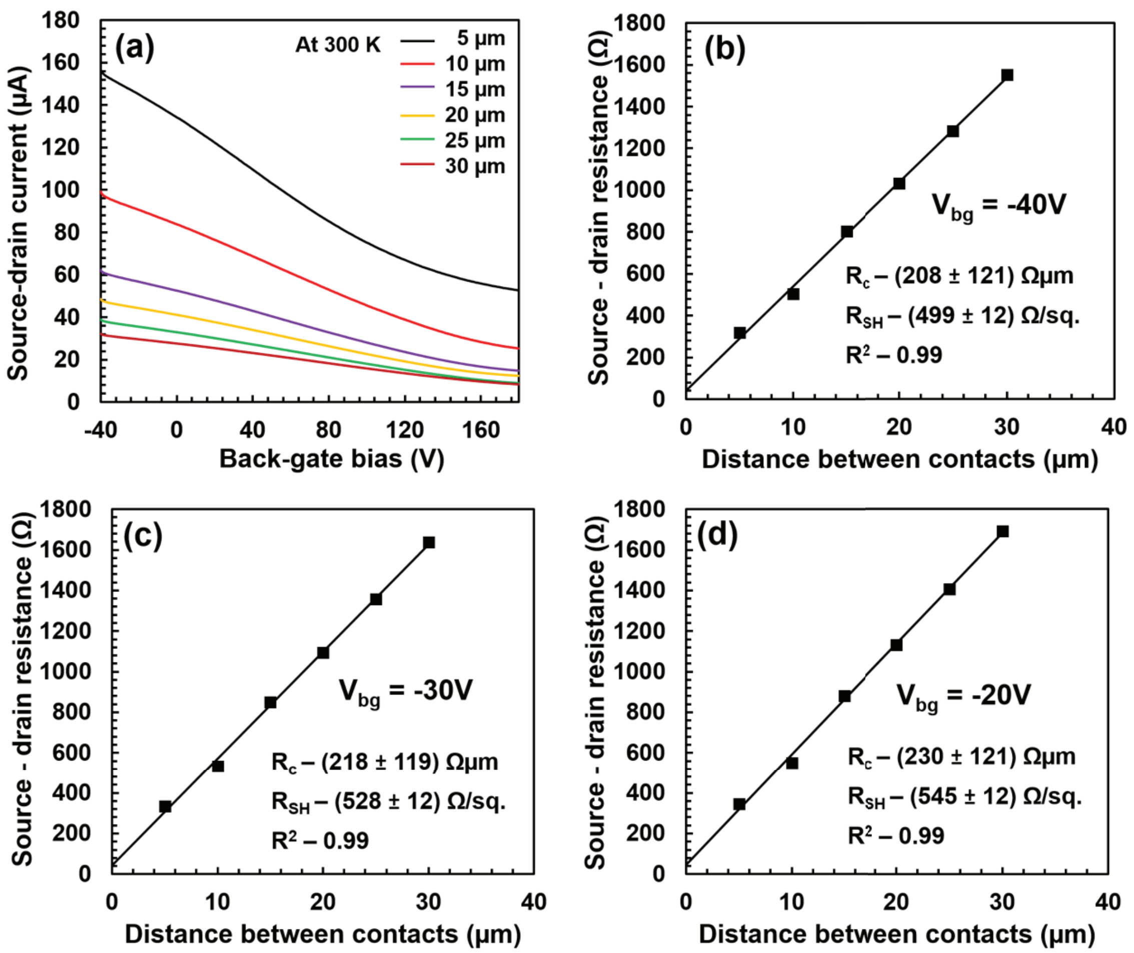

Figure S3: **Experiments on palladium contacts: a)** Transfer characteristics of a TLM structure with palladium contacts on graphene as a function of the back-gate voltage V_{bg} for different spacing between contacts. Temperature is 300 K and the voltage applied between the contacts is 50 mV. **b,c,d)** Total resistance between source and drain contacts as a function of spacing between contacts for back-gate voltage V_{bg} of -40 V (b), -30 V (c), -20 V (d). Squares are experimental data. From the least mean square fit (line), the contact resistance R_c from the Y-intercept and the sheet resistance $R_{\rm sh}$ from the slope of the line are extracted.

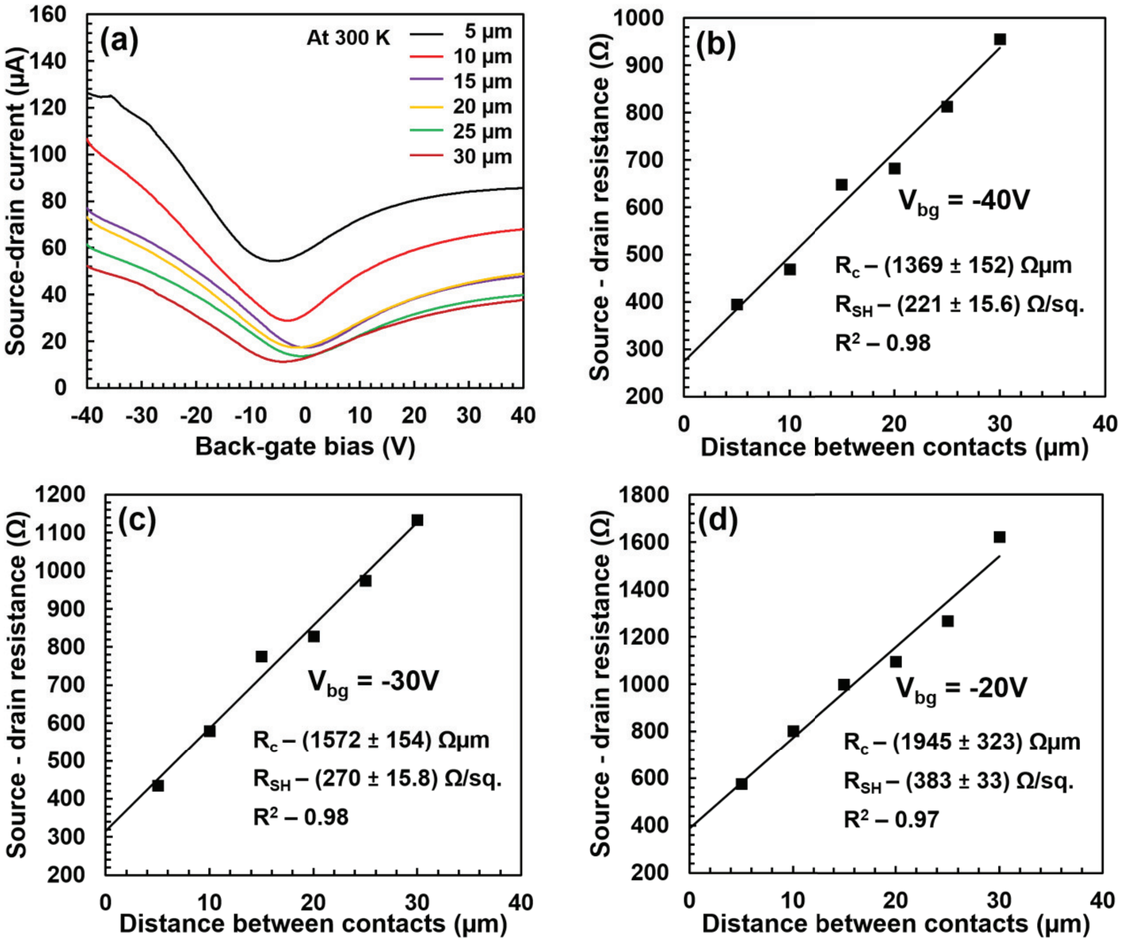

Figure S4: **Experiments on platinum/gold contacts: a)** Transfer characteristics of a TLM structure with platinum/gold contacts on graphene as a function of the back-gate voltage V_{bg} for different spacing between contacts. Temperature is 300 K and the voltage applied between the contacts is 50 mV. **b,c,d)** Total resistance between source and drain contacts as a function of spacing between contacts for back-gate voltage V_{bg} of -40 V (b), -30 V (c), -20 V (d). Squares are experimental data. From the least mean square fit (line), the contact resistance R_c from the Y-intercept and the sheet resistance R_{sh} from the slope of the line are extracted.

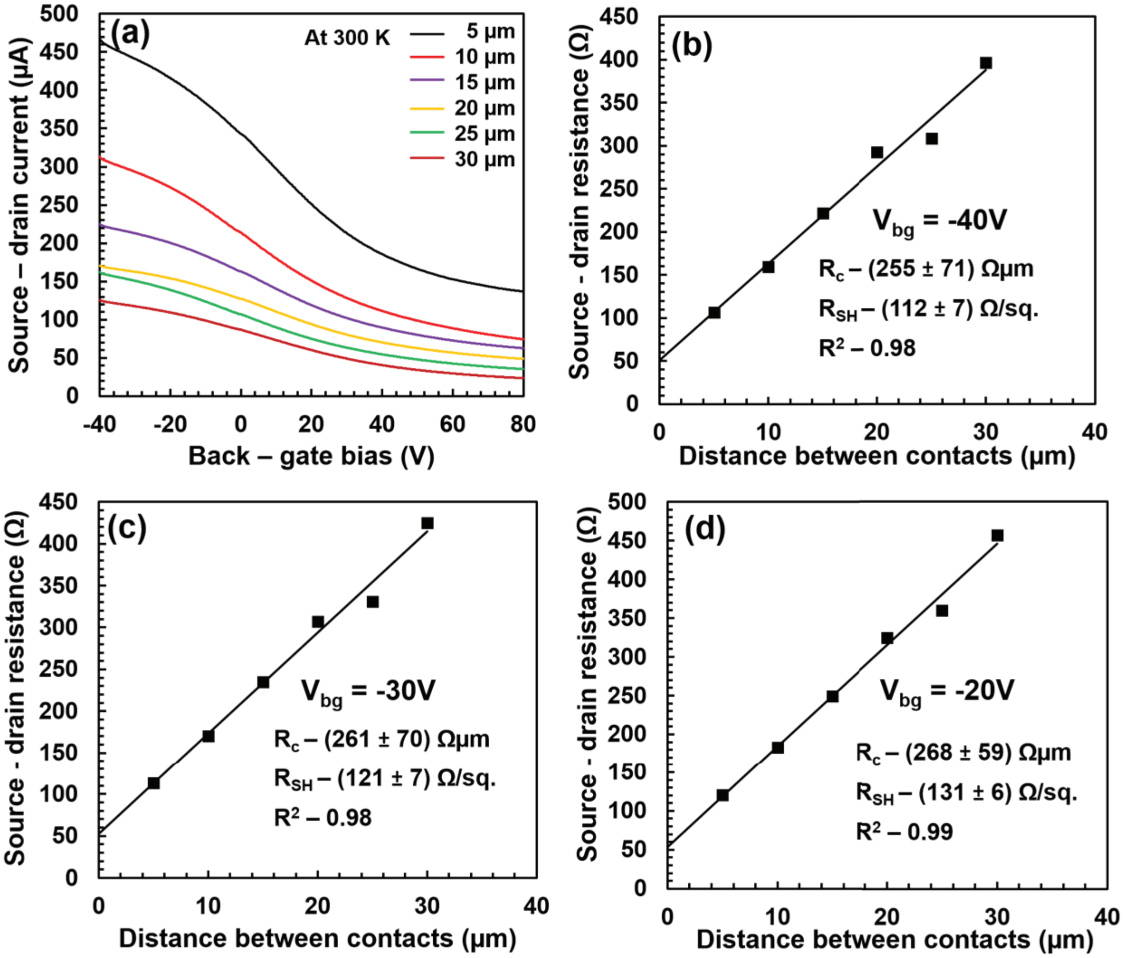

Figure S5: **Experiments on copper contacts: a)** Transfer characteristics of a TLM structure with copper contacts on graphene as a function of the back-gate voltage V_{bg} for different spacing between contacts. Temperature is 300 K and the voltage applied between the contacts is 50 mV. **b,c,d)** Total resistance between source and drain contacts as a function of spacing between contacts for back-gate voltage V_{bg} of -40 V (b), -30 V (c), -20 V (d). Squares are experimental data. From the least mean square fit (line), the contact resistance R_c from the Y-intercept and the sheet resistance R_{sh} from the slope of the line are extracted.

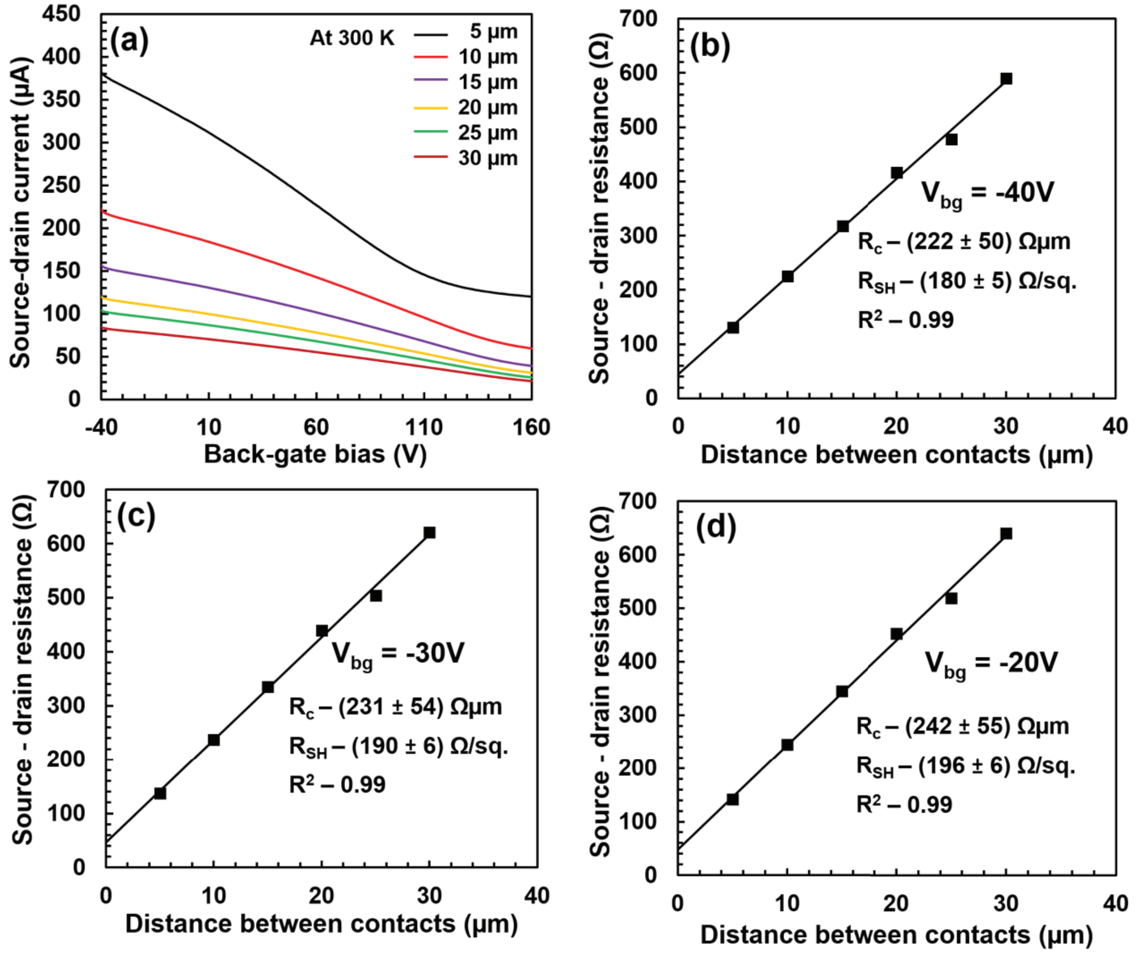

Figure S6: **Experiments on gold contacts: a)** Transfer characteristics of a TLM structure with gold contacts on graphene as a function of the back-gate voltage V_{bg} for different spacing between contacts. Temperature is 300 K and the voltage applied between the contacts is 50 mV. **b,c,d)** Total resistance between source and drain contacts as a function of spacing between contacts for back-gate voltage V_{bg} of -40 V (b), -30 V (c), -20 V (d). Squares are experimental data. From the least mean square fit (line), the contact resistance R_c from the Y-intercept and the sheet resistance R_{sh} from the slope of the line are extracted.

Ni nanowires with ultrasoft pseudopotentials *Phys. Rev. B* **2004**, 70, 045417

(S3) Choi, H.J.; Ihm, J.; Ab initio pseudopotential method for the calculation of conductance in quantum wires *Phys. Rev. B* **1999**, 59, 2267

(S4) Ji, X.; Zhang, J.; Wang, Y.; Quian, H.; Yu, Z.; A theoretical model for the contact ressitance using a DFT-NEGF method *Phys. Chem. Chem. Phys.* **2013**, 15, 17883

(S5) Liu, H.; Kind, H.; Ohmo, T.; Contact effects of nickel and copper on electron transport through graphene *Phys. Rev. B* **2012**, 86, 155434

(S6) Stokbro, K.; Engelund, M.; Blom, A.; Atomic-scale model for the contact resistance of the nickel-graphene interface *Phys. Rev. B* **2012**, 85, 165442

(S7) Girit, C..; Meyer, J.C.; Erni, R.; Rossell, M.D.; Kisielowski, C.; Yang, L.; Park, C.-H.; Crommie, M.F.; Cohen, M.L.; Louie, S.G.; Zettl, A.; Graphene at the edge: Stability and Dynamics *Science* **2009**, 323, 1705-1708

(S8) Giovanetti, G.; Khomyakov, P.A.; Brocks, G.; Karpan, V.M.; van den Brink, J.; Kelly, P.J.; Doping Graphene with Metal Contacts *Phys. Rev. Lett.* **2008**, 101, 026803

(S9) Bengtsson, L.; Dipole correction for surface supercell calculations *Phys. Rev. B* **1999**, 59, 12301

(S10) Cheng, D.; Barcaro, G.; Charlier, J.C.; Hou, M.; Fortunelli, A.; Homogeneous Nucleation of Graphitic Nanostructures from Carbon Chains on Ni(111) *J. Phys. Chem C* **2011**, 115, 10537-10543

(S11) Kwon, S.-Y.; Ciobanu, C.V.; Petrova, V.; Shenoy, V.B.; Baren, J.; Gambin, V.; Petrov, I.; Kodambaka, S.; Growth of Semiconducting Graphene on Palladium *Nano Lett.* **2009**, 9, 3985-3990

(S12) Gong, C.; McDonnell, S.; Qin, X.; Azcatl, A.; Dong, H.; Chabal, Y.J.; Cho, K.; Wallace, R.M.; Realistic Metal-Graphene contact structures *ACS Nano* **2014**, 8, 642-649

(S13) Ma, B.; Gong, C.; Wen, Y.; Cheng, R.; Cho, K.; Sham, B.; Modulation of contact resistance between metal and graphene by controlling the graphene edge, contact area, and points defects: An ab initio study *J. Appl. Phys.* **2014**, 115, 183708

(S14) Franciosi, A., Van de Walle, C.G.; Heterojunction band offset engineering *Surf. Sci. Rep.* **1996**, 25, 1-140

(S15) Montgomery, D. C., Runger, G. C.; *Applied statistics and probability for engineers* 6th Edition, Wiley, 2014, p. 447-448
